# Direct Marketing Experiences Among Individuals With Current and Lifetime Gambling Disorder

**DOI:** 10.3389/fpsyg.2020.01957

**Published:** 2020-08-05

**Authors:** André Syvertsen, Ståle Pallesen, Eilin Kristine Erevik, Rune Aune Mentzoni

**Affiliations:** ^1^Department of Psychosocial Science, University of Bergen, Bergen, Norway; ^2^Norwegian Competence Center for Gambling and Gaming Research, University of Bergen, Bergen, Norway

**Keywords:** gambling promotions, advertising, problem gambling, online gambling, Norway

## Abstract

Gambling providers use varied and complex marketing techniques, including marketing that targets the individual directly. Previous research indicates that individuals with gambling disorder are disproportionately influenced by gambling marketing, however, very few studies have examined gamblers’ experiences with direct marketing. The current exploratory interview study examined experiences with direct gambling marketing among 12 individuals with either current (*n* = 5) or lifetime (*n* = 7) gambling disorder. A broad research question was employed encompassing experiences with different types of direct marketing and corresponding attitudes, influences, and interactions. The interview data were analyzed with thematic analysis using an inductive approach, and the participants reported extensive and varied experiences with direct marketing. Two overarching themes, with two and four subthemes, respectively, were identified. The overarching themes showed that marketing experiences were intimately connected with participants’ gambling behaviors and their relationships to their own problems. Overall, direct marketing was experienced as an interactive form of marketing with individually tailored promotions such as free gambling credits, bonuses, and special gifts. Some promotions were experienced as personal, while others were experienced as mass produced. Direct marketing was in some cases experienced as predatory and was reported to hamper the participants’ ability to cope with their gambling disorder. Participants’ attitudes toward direct marketing varied as a function of the participants’ engagement in gambling. During periods of active gambling, direct marketing was experienced as beneficial and positive as the participants took advantage of the offers or actively manipulated how the offers were made. In contrast, when attempting to reduce/abstain from gambling, the participants experienced direct marketing as aggressive, and they reported making considerable efforts to try to limit it. Direct marketing was experienced as a trigger for gambling urges and was reported to induce a conflict between gambling and abstinence. Directly marketed promotions are discussed in relation to ecological factors of access and availability that form a basis for the development of gambling disorder, and variations in experiences are related to stages of change in gambling disorder. Implications for treatment are discussed where the current findings suggest that coping with marketing should be addressed in treatment.

## Introduction

Gambling presents little or no harm for most people, while a minority of people experience negative consequences and loss of control. In such cases the person might, among other things, lose increasing amounts of money, acquire debts, commit illegal acts to finance gambling, and experience relationship or career-related losses due to their gambling behavior (Shaffer and Martin, 2011). Severe gambling problems may fulfill the diagnostic criteria for gambling disorder (GD), as described in the most recent edition of the Diagnostic and Statistical Manual for Mental Disorders (5th version, American Psychiatric Association, 2013) and the recently published 11th edition of the International Classification of Diseases and Health Problems (World Health Organization, 2018).

Today, gambling opportunities are ubiquitous, spearheaded by the introduction of online gambling which is marketed in complex and varied ways (Gainsbury, 2012; Hing et al., 2014). Studies generally suggest positive associations between exposure to gambling marketing and GD (Binde, 2014; Newall et al., 2019). The association between GD and exposure to gambling marketing might be explained by disordered gamblers being more attentive to such marketing or being more likely to receive it due to their gambling history, or by exposure to gambling marketing contributing to the development of GD. The self-rated influence of gambling marketing is higher among those with GD and greater influence is typically reported in the form of increased gambling, gambling urges, or thoughts about winning in response to the marketing (Binde, 2014; Newall et al., 2019).

Promotional marketing differs from traditional basic marketing (Van Waterschoot and Van den Bulte, 1992). While basic marketing aims to increase wanting and brand awareness, promotional marketing aims to trigger action. Gambling shirt sponsorships of English football teams by gambling companies has been increasing over the last decade and represents a form of basic marketing (Lopez-Gonzalez and Griffiths, 2017). Promotional marketing can include sign-up bonuses, deposit bonuses, cash back, and free gambling credits to new and/or established customers (Hing et al., 2015). Gamblers report influence from promotions in the form of increased gambling involvement, reduced perception of risk, and increased impulsive betting (Hing et al., 2014, 2017, 2019; Deans et al., 2017). Further, several studies have found greater self-reported influence from promotional marketing compared to basic marketing among people with GD (Thomas et al., 2012; Hing et al., 2017), but this has not been consistently shown (Hing et al., 2019). Hing et al. (2014) found that disordered gamblers reported salient experiences related to promotional marketing. In their study, treatment-seeking gamblers reported that promotional marketing triggered gambling sessions and undermined efforts to reduce or stop gambling. Promotions were further reported to lead to longer gambling sessions through increased availability of gambling funds. Participants also experienced promotions that were tailored to their gambling history. For instance, treatment-seeking participants who abstained from gambling reported receiving promotions that they perceived as aimed at getting them to resume gambling.

Gambling marketing, including promotions, can be communicated directly to the individual by employing direct communication channels such as phone calls, text messages, and emails, i.e., direct marketing. Russell et al. (2018) examined direct marketing in an ecological momentary assessment study of self-reported gambling behavior and found that receiving emails was associated with increased betting intention, while receiving texts was associated with greater likelihood of betting and increased betting amounts (Russell et al., 2018). Combining promotional marketing with the use of direct communication channels is likely to be an especially potent form of influence. People with GD spend and lose large amounts of money, and this can be assumed to make them attractive targets for such marketing and highlights the need to examine direct marketing experiences within this group (Orford et al., 2013). Studies specifically examining direct marketing are lacking, a likely reason for this is that direct marketing, by virtue of being available only to selected recipients, is harder to study (for an exception, see Russell et al., 2018).

The current study is based on a series of semi-structured interviews concerning direct marketing with a sample consisting of individuals with current or lifetime GD. Differentiation between current and lifetime GD allows for nuances regarding how direct marketing is experienced by individuals in different phases of GD. Given the findings that marketing experiences are influenced by GD characteristics, this seems to be an important distinction. The study was guided by the following research questions:

What experiences do individuals with current or lifetime GD have with gambling-related direct marketing with regards to:

(1) the types of direct marketing experienced and their attitudes toward these types?
(2) their interaction with direct marketing?
(3) the perceived influence from direct marketing?

The research questions were understood in a broad manner. Types of direct marketing refer to form, content, or general characteristics. Interaction refers to any form of activity involving direct marketing, including actions in response to direct marketing offers and exchanges between gamblers and gambling company representatives. Finally, influence refers to the perceived influence on gambling behavior and other areas deemed important by the participant. The research questions capture several aspects that Binde (2014) argues should be given priority in gambling marketing research, namely content, attitudes, and influence. Further, qualitative studies have been argued to be especially valuable in providing insights into possible mechanisms of influence in gambling marketing and to be appropriate for initial exploratory research on this topic (Binde, 2008, 2014; Hing et al., 2014).

There are various regulations for marketing across different nations and provinces (Planzer et al., 2014). The current study was conducted in Norway in which the state-owned gambling providers Norsk Tipping and Norsk Rikstoto have a monopoly on lotteries/sports betting and horse betting, respectively (Rossow and Hansen, 2016). Norsk Tipping and Norsk Rikstoto may only directly market general information about games to consenting customers (e.g., reminders about upcoming lotteries) (Ministry of Agriculture, 2014). Unregulated gambling operators are prohibited from marketing in Norway, but this is circumvented by advertising on television channels that are aimed at Norwegians (i.e., marketing using Norwegian language and content) but which are broadcast from abroad. Consequently, unregulated online gambling operators are well known and easily accessible for Norwegian customers.

## Materials and Methods

### Study Design

Individual semi-structured face-to-face interviews were conducted taking a phenomenological approach that prioritizes participants’ own perspectives and experiences (Creswell and Poth, 2016). Participants’ accounts were treated as reflecting their inner psychological experiences, which is a theoretical position referred to as realism (Madill et al., 2000).

### Recruitment and Sample Characteristics

Purposive criterion sampling was used to recruit individuals with either current or lifetime GD (Patton, 2002). Participants were recruited through Norwegian self-help groups for GD. Organizers of the self-help groups were contacted, and they forwarded study information to attending members. Those interested in participating provided their telephone numbers and were contacted by the project leader (AS). Information about the study was repeated, and a time and place for the interviews were agreed upon. Questions related to the DSM-5 criteria for GD were asked to verify that the participants suffered for this disorder either currently or lifetime (American Psychiatric Association, 2013). In total, 12 participants were recruited to ensure a sufficient sample size to identify patterns across the data set, while also keeping a focus on individual experiences in line with our phenomenological approach. We aimed to reach the recommended sample size for small scale interview studies that employ thematic analysis on participant experiences, i.e., between six to ten participants (Braun and Clarke, 2013). The decision to include 12 instead of ten participants was made in order to account for possible participant attrition.

A semi-structured interview guide was developed by AS, SP and RM (see Supplementary Materials). The development of the interview guide was influenced by the current lack of research on direct marketing. As such, we prioritized open-ended and broad questions covering types of direct marketing, possible influences from direct marketing and interaction. The questions were developed based on existing knowledge of types of gambling marketing and communication channels (Binde, 2014; Newall et al., 2019) formulations regarding topics that should be explored in research on gambling marketing (i.e., content, attitudes and influence). We included descriptive, contrast and evaluative questions (Willig, 2013). Descriptive questions concerned general accounts of their experiences with direct marketing. Contrast questions involved comparisons between participants’ experiences with direct versus indirect marketing. Evaluative questions were related to participants’ feelings toward and potential influence from direct marketing. A range of interviewing techniques were used in the interviews (Brinkmann and Kvale, 2015). Participants’ accounts were followed up with probing questions for greater detail and specifying questions for clarity. Silence was used to allow for greater reflection in the interview. Interpretive questions were also used to summarize participants’ accounts and strengthen mutual understanding throughout the interview.

Sampling and interviewing took place during the fall of 2017. The interviews were conducted in Norwegian and lasted between 28 and 51 min with an average of 37 min. Participants were compensated 250 NOK (approximately 25 €) for their participation. The final sample consisted of 12 individuals (including two women) who met the criteria for either current (*n* = 5) or lifetime (*n* = 7) GD. Participants’ ages ranged from 29 to 55 years with an average of 41 years (*SD* = 7.76 years).

### Ethics Statement

The study was approved by the Regional Committee for Medical and Health Related Research Ethics in Western Norway (no. 2017/1172). All participants provided written informed consent in accordance with the Declaration of Helsinki. Written, informed consent was obtained from the participants for the publication of any potentially identifiable data included in this article. Specifically, information on age, gender and gambling history and use of verbatim quotes.

### Data Analysis

The data were analyzed for meaningful patterns in relation to the research questions through thematic analysis (Braun and Clarke, 2006, 2013; Clarke and Braun, 2018). In thematic analysis, the identified patterns, called themes, can be categorized based on either an inductive approach (where the data are given priority) or a theoretical approach (where predetermined analytical categories are given priority). In this study the inductive approach was used.

Thematic analysis consists of the following six phases: (1) familiarizing yourself with the data, (2) generating initial codes, (3) searching for themes, (4) reviewing themes, (5) defining and naming themes, and (6) producing the report (Braun and Clarke, 2006, 2013). AS conducted the main analysis of the data. The themes and supporting data were credibility checked by RM and a second researcher from the University of Bergen. Inclusion of several researchers in the credibility check of themes is in line with the recommendations of Elliott et al. (1999). Credibility checking featured throughout data analysis with repeated discussions of themes and their representation in codes. We aimed at satisfying Braun and Clarke (2006) checklist of criteria for good thematic analysis.

Data analysis began at the phase of familiarization. The audio-taped interviews were transcribed verbatim but without detailed paralinguistic features. That is, they included what was said and by whom, including all words and verbal utterances (e.g., “ehh”), but not non-vocal signals (e.g., body language). Significant paralinguistic features such as laughter and long pauses were also transcribed, albeit not at the same level of detail. Initial notes and ideas for themes were made during this phase, and familiarization continued with repeated readings of the transcripts.

Initial codes were generated with the help of the software program NVivo 11. Complete semantic coding was done as the interview transcripts were worked through. The coding was complete in that it included all aspects that were relevant to the research questions, and it was semantic in that it mapped onto what was said with minimal interpretation at this stage. An example code is “DM content - bonuses” referring to any talk of directly marketed bonuses. Transcripts were worked through multiple times because the initial coding highlighted aspects that were relevant to recode. After the collapsing of similar codes, 72 unique codes remained.

Searching for potential themes involved identifying meaningful patterns across codes. Themes were identified based on a central organizing concept—a coherent idea. Subthemes contained distinctive patterns within these themes, and these were useful if a theme’s central organizing concept played out in different ways. A single code could appear in more than one theme if it was related to the central organizing concept of the different themes.

The next phase involved reviewing themes. This was conducted by AS, RM and a third researcher from the University of Bergen. Themes were changed or combined if they did not cohere well within themselves or overlapped too much with others. Reviewing involved two phases. The first phase involved examining coded extracts and deciding if their content was adequately represented in the themes that were identified. The second phase involved going over all data items (interviews) to ensure that everything relevant to the research questions was represented in the themes. An example of credibility checking at this phase concerned a discussion of the central organizing concept of the second overarching theme “psychological distance to gambling determined the direct marketing experiences.” One alternative suggestion was that (lack of) abstinence to gambling would be a meaningful idea connecting reported experiences. However, it was ultimately decided that a participant’s psychological distance to gambling better captured participant’s accounts because even participants who struggled to abstain from gambling could express rejection of gambling marketing, suggesting that gambling was not consistent with their ideal selves. Examination of interview extracts were instrumental in resolving this discussion.

Themes and subthemes were then defined and named. Definitions included clear statements about the central organizing concept and statements regarding the overall relationship between the themes and the research questions.

Finally, a unified report was created. The first paragraph in the theme and subtheme sections contained a definition of the corresponding central organizing concept. The themes primarily consisted of meaningful parts of the data, and their relevance to the research questions was prioritized over their frequency in the data. Despite this, some quantifying language was also used to give a sense of the frequency of different experiences. The following terms were thus used to refer to the number of participants: “a few” 1–3, “several” 4–5, “many” 6–8, “most” 9–11, and “all” 12.

## Results

**FIGURE 1:**
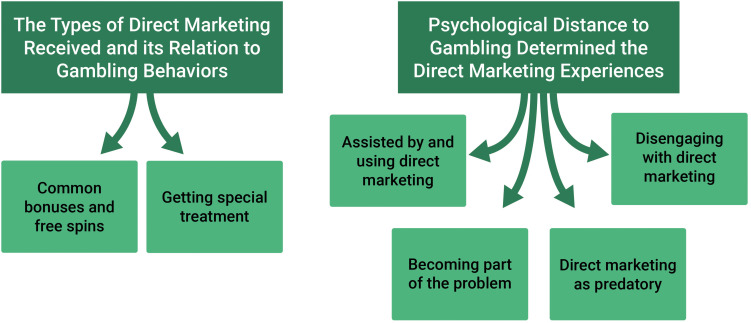
A thematic map illustrating two overarching themes with two and four subthemes, respectively, that captures participants’ experiences with gambling-related direct marketing.

Two overarching themes with two and four subthemes, respectively, were identified. The first theme – “The types of direct marketing received and its relation to gambling behaviors” – covered participants’ experiences with types of direct marketing. That is, what types of direct marketing received and under which circumstances and channels they received it. The second theme – “Psychological distance to gambling determined the direct marketing experiences” – covered gamblers’ experiences with direct marketing interactions, influence, and attitudes. That is, how the direct marketing participants received was reacted to and perceived. Figure 1 provides a thematic map of the identified themes and subthemes.

### The Types of Direct Marketing Received and Its Relation to Gambling Behaviors

This theme captures participants’ experiences with different types of direct marketing, as well as showing how direct marketing was experienced as both similar to and different from indirect marketing. The central organizing concept of this theme is the relation between gambling behaviors and the type of marketing received. Experiences with some types of direct marketing were found to independent of gambling behaviors (see section “Common Bonuses and Free Spins”) while other types of direct marketing were dependent on level of engagement with gambling companies (see section “Getting Special Treatment”).

#### Common Bonuses and Free Spins

This subtheme captures how certain types of direct marketing were experienced as especially common. This included promotions such as bonuses, free spins, and small amounts of free credit.

When participants were asked to freely describe the content of the direct marketing, promotions in the form of bonuses and free spins comprised the most salient parts of their descriptions. Not only were they mentioned by all participants, but they were also often implicated throughout the entire interview. The contents and terms of these promotions were described in a matter-of-factly and familiar way, as illustrated in the following quote:

No, it’s always the same way, it’s just, go make yourself an account or deposit money and get the 5-fold (bonus for the deposited amount), then you have to play for probably 30-fold (of the deposited amount) to be able to withdraw the winnings, right. (46-year-old woman, lifetime GD).

Such promotions were considered by the participants to be mass communicated and generic. They mostly concerned casino games and were mainly communicated by email, although some mentioned texts and postal promotions as well. For example, while contrasting different channels for sending the promotions, one participant said: “[…] *emails are more, like, general. They send out loads of them to everybody*” (*55-year-old woman, lifetime GD*).

When there was low interactivity, the direct marketing content mirrored that of indirect marketing. Similar offers were communicated by both unknown gambling companies and familiar ones. The following quote captures such similarities:

I don’t see it as that different actually [talking about indirect marketing]; it’s mostly free spins and bonuses, get something free for depositing money. That’s usually what it is, it’s usually the casinos, but then there’s football, so sports betting, but that’s a whole other world. In that case there’s very little direct advertising. In fact, what gets seen on TV is very similar [comparing it to direct marketing], except for gifts and stuff, but that wasn’t very often. (33-year-old man, lifetime GD).

As the above quote also illustrates, direct marketing experiences were related to game types. Some engaged exclusively with electronic slot machines or other forms of casino games such as blackjack or roulette. Others also engaged in sports betting, stock trading, and poker. However, regardless of whether the participant played mainly casino games or not, direct marketing for casino games was a typical experience and was even expected among the participants.

#### Getting Special Treatment

Participants described how increased engagement with gambling companies led to changes in promotions such as bonuses and free spins and that the participants also received new forms of direct marketing. Turnover requirements related to bonuses were lowered, and free credit was increased. A few had experiences with custom arrangements in which they were returned a fixed percentage of losses over a given period, i.e., “cash back.” One participant with high engagement in gambling compared the different agreements he had experienced, noting that the percentages and time periods varied: “[…] *I get cash back on, if I, let’s say I’ve lost hundred, hundred thousand* [*NOK, approximately 10,000* €] *in 3 days, then they would probably give me fifteen thousand, five thousand back immediately*” (*29-year-old man, current GD*).

Losing large amounts of money with certain casinos could result in substantial amounts of free credit too, as shown in the following quote (the context of this quote suggests that the participant refers to losses with an online casino):

[…] If I was very active and had spent many hundreds of thousand [NOK] at a casino then it was seemingly often that they then added money as time went by. First two thousand kroners, three thousand kroners, five thousand kroners, and then they keep going. (34-year-old man, current GD).

Phone calls were reported less frequently compared to other channels of direct marketing and mostly involved instances in which the participant engaged heavily with a given company. Direct marketing through phone calls might involve simple invitations to participate in marketing surveys or information about bonus offers. However, experiences with phone calls more often entailed a more personal quality and involved tailored or specific offers. Participants stated that they were addressed by name and that the callers referred to special events such as holidays. One participant recalled how the caller referenced his upcoming birthday, asking what he wished for. Participants described interactions in which the callers generally emphasized that the participant was a special and highly valued customer.

Many participants had experienced what can be regarded as special and, in some cases, extravagant offers. This included sponsored dinners with other valued customers, attendance to parties with celebrity ambassadors, sponsored trips to foreign countries, free access and means to attend gambling events, sponsored gambling cruises, and items such as guitars, gift cards, shirt cuffs, pens, and candy. Such special offers were reported by seven participants, and their accounts revealed that they had high engagement with the company in question. The participants also interpreted this to be due to their loyalty. One participant who received both dinner and party invitations from her gambling company of choice surmised: “*No, I just assume that I used a lot of money to play so I entered into some sort of VIP thing*” (*55-year-old woman, lifetime GD*). How participants were influenced by and interacted with special offers depended on how the participants related to their own gambling problems (see “Becoming Part of the Problem”).

Many of the participants also spoke of direct marketing being perceived as more dynamically linked with their gambling patterns. Here marketing was tailored not only to the strength of their engagement, but also to nuanced parts of their gambling patterns. They were left with the impression that they received bonus offers when actively depositing money and gambling and received free credit when they became inactive. The descriptions of absence-induced promotions mostly involved phone calls, although some mentioned emails or spoke of it more generally like this participant: “[…] *in some cases they will actually go as far as to give you free money in your gambling account just to get you going again*” (*44-year-old man, current GD*).

### Psychological Distance to Gambling Determined the Direct Marketing Experiences

This theme captures how experiences with direct marketing were found to be related to how psychologically distanced the participants were to their own gambling problems and to gambling overall. Accounts of experiences with direct marketing took the form of “back then versus now” among participants with lifetime GD. This was also the case for two participants with current GD who stated that they had now clearly distanced themselves from gambling. The three remaining participants with current GD gave accounts that were suggestive of ambivalence (see below). It appeared that it was the rejection of gambling more than the status of their diagnosis or their length of abstinence that characterized the way the participants reported their experiences with direct marketing.

Participants with lifetime GD and those with current GD who made a clear separation between “then and now” reported experiencing direct marketing and gambling marketing overall as predominantly positive during active gambling, which was contrasted to a predominantly negative experience now that they did not gamble. Reports were more conflicting in the “ambivalent group” of those with current GD. The first two subthemes reflect active play periods and periods when gambling was psychologically close, while the last two contain experiences related to periods where the participants rejected marketing and gambling overall.

#### Assisted by and Using Direct Marketing

All participants had taken advantage of direct marketing offers during their active play periods. During play periods, these offers were experienced as welcome assistance that became interspersed with their gambling activities. This subtheme was supported by direct accounts as well as indirectly through accounts of interactions with such marketing. The subtheme captures the way that offers from direct marketing were used.

People interacted differently with direct marketing offers. Half of the sample made use of them as they came, while the other half actively sought out more or better offers as well. Collectively these descriptions indicated that some marketing techniques allowed them to sustain gambling beyond what would otherwise be possible. Bonus offers extended gambling funds, and free spins and free credit allowed for gambling even when the person was broke. One participant stated succinctly: “*They give me gambling money when I don’t have any myself*, [*laughs*]” (*55-year-old woman, lifetime GD*).

A few of those who did not seek out offers still made extensive use of the offers they received, as can be illustrated in the following quote:

If I didn’t have any money then I made use of all the offers with free spins […]. If I had money, then I made use of bonus deposits that I could get; that way I could get the most to play for at any given time. (37-year-old man, lifetime GD).

The other half who actively sought out offers were proactive in several ways. This could involve taking advantage of how marketing was tailored to their activity by deliberately switching between companies to get better offers. A few would negotiate better offers with company representatives. One participant recalls getting a call from a representative: “*Yes, I tried to keep him warm, tried to get him to give me an even better offer because I didn’t think the offer was especially good*” (*44-year-old man, current GD*). Another stated that he had reached out to company representatives multiple times in order to get better cash back terms.

#### Becoming Part of the Problem

All but one participant explicitly said that direct marketing influenced them during active play periods. Further, as noted above, all participants (including the participant who did not report being explicitly influenced) made use of direct marketing, which also presupposes some form of influence. The one participant who denied being influenced stated that his gambling had been so extensive that despite actively using direct marketing, he believed that the absence or presence of direct marketing would have made no difference. Influence from and interaction with direct marketing was reported to be intimately linked to GD, a link that is captured by this subtheme.

Participants reported influence from direct and indirect marketing in the form of such marketing eliciting gambling and gambling urges. Most emphasized stronger influences from direct marketing which were experienced to include more attractive offers and to make them feel special. One participant contrasted between direct and indirect marketing in this way: “*What comes directly to you is more tempting because people are treated differently, people are different*” (*29-year-old man, current GD*). There was one exception in which a participant said that television commercials had a greater effect because of the vivid sounds and imagery that triggered gambling urges. A few participants noted that direct marketing could also trigger gambling or gambling urges indirectly by reminding the participant of gambling in general even if the participant did not make use of the specific offer.

Free credit and free spins were emphasized by many participants. Such offers were highlighted as causing increased gambling or triggering gambling urges. This was detailed in various ways. One participant recalled: “*It was like a trigger then, or the way I thought about free spins, it’s like I don’t actually use any money so it’s not dangerous. I could just play those free spins*” (*37-year-old man, lifetime GD*). Another participant with current GD echoed the sentiment that such offers seemingly presented no downsides, but recognized cases in which he continued to gamble with his own money afterward. Yet another person with current GD explained that he struggled with the knowledge that he had readily available gambling funds waiting for him – this made him feel restless and triggered hopes of recovering losses. Two participants reported valuing gambling funds more than special offers of similar or higher monetary value that could not be used directly for gambling, which indicates gambling salience among these participants. One participant wanted money instead of an expensive guitar or a trip, and another chose more gambling funds over a sponsored trip abroad:

They offer you a trip, it’s a bit funny, they call and ask if you want a trip to London to watch football or if you would like so and so amount of money [deposited] into your [gambling] account… You would have said football trip to London, right? I’d say deposit money into my gambling account. (33-year-old, man, lifetime GD).

Several participants reported that they tried to conceal their direct marketing use along with their overall gambling behavior during active periods. None of the participants who received special offers to attend sponsored dinners, parties, cruises, or trips to foreign countries accepted these offers. Probing questions revealed that one reason was the concern that others would learn of their gambling if they attended. They worried that gambling companies would actively use their dinner or party attendance for more public marketing campaigns, possibly exposing them. A few said they made efforts to conceal all instances of direct marketing. They would talk about making efforts to hide texts and emails and to destroy postal letters so that significant others would not see them. At the same time, they could be reluctant to attempt to exclude themselves from marketing because they also wanted to make use of the offers.

Many participants, comprising both those with current GD and lifetime GD, stated that marketing could trigger unpleasant memories related to gambling and the different kinds of losses that they associated with it. As one participant stated: “*I feel like it, it triggers extra aggression and problems in the family, and that kind of thing* […] *That’s hard on both the one playing and the rest of the family*” (*41-year-old man, lifetime GD*).

#### Direct Marketing as Predatory

This subtheme captures the finding that many participants experienced direct marketing as negative and/or incessant and that such experiences were more common when the persons had distanced themselves psychologically from gambling.

When participants with lifetime GD talked about their current experiences and attitudes toward direct marketing, it was mostly in negative ways. This was also the case with two participants who had current GD but who had distanced themselves psychologically from gambling. Their statements indicated a realization of the “true nature” of direct marketing techniques; that these offers had been manipulative or even scams all along. Communication that was previously seen as good customer service was now regarded as strategies meant to disproportionately benefit the gambling companies. One participant spoke of the marketing in this way:

[…] see here you have three thousand [NOK], so if you come back you’re going to play for forty [thousand NOK], then that’s not so good for me, but it’s probably very good for them, even though it seems like a very good offer for me (42-year-old man, lifetime GD).

Bonuses were perceived as trickery because the turnover requirements were seen as very high, making net gains unlikely. Free spins were also seen as being of little importance. Negative accounts of promotions were also mentioned by the remaining participants with current GD who were less psychologically distanced from gambling, although their grievances highlighted other aspects. Rather than emphasizing morality, these three participants put more emphasis on the unattractiveness of the offers – that they were bad offers. As stated by one participant: “*They’re uninteresting because you get very little bonus and there’s a big turnover requirement for being able to withdraw money*” (*44-year-old man, current GD*).

Many stated that they had or were experiencing overwhelming amounts of direct marketing, which made them angry or annoyed, and they described the marketing as aggressive and constant. A few participants emphasized that indirect marketing in the form of TV commercials added to this oversaturation. Most could not recall giving permissions for direct marketing, but also stated that they might have done so carelessly when signing up for gambling sites. However, all participants had experienced cases without giving permission as well, evident by the fact that they received large amounts of direct marketing from unknown gambling companies. This was also the case for the participants who had been abstinent for a long time. A few added that they thought gambling companies carelessly shared their personal contact information with other companies.

The majority of those who had attempted to exclude themselves from gambling sites had stopped receiving marketing from those specific gambling sites/companies, but there were several exceptions. One participant said she had gotten a call from a gambling representative months after she had excluded herself. The representative had offered her 6,000 NOK deposited into her previously locked gambling account without any conditions for use. Another participant was phoned by the company a year after she excluded herself, and the gambling representative emphasized that the participant had been a valued customer and the participant was asked if the gambling company could offer her anything to make her return to the gambling site. A participant that received unwanted direct marketing reported difficulties making it stop:

So I have called them and asked if they can delete me from the list, but they say that’s not possible because you have to go into your account to stop the text communication, but my account’s locked so that’s impossible (37-year-old man, lifetime GD).

#### Disengaging With Direct Marketing

This subtheme captures how interactions with direct marketing changed when the participants had stopped or were trying to stop gambling. Participants with current GD expanded upon the influences reported in the subtheme “Becoming part of the problem” with statements about how they coped with these issues. Some strategies were related to direct marketing specifically, while others concerned all forms of gambling marketing.

Participants with lifetime GD and long abstinence stated that direct or indirect marketing exerted little current influence in terms of eliciting gambling urges or increasing self-perceived risk of relapse. In one exception, a participant stated that marketing could still trigger gambling urges, which he handled by playing social gambling games with fake money. Another participant recalled relapsing due to a combination of direct and indirect marketing during a previous attempt to abstain from gambling. Current influence was generally perceived in a way that indicated that marketing had lost its significance, leaving just the negative attitude noted in the subtheme above. Thus, the gamblers did not speak of any need to actively exercise coping behavior anymore. Even substantial offers were ineffective, and in the following quote the participant had been offered a cruise trip and was asked if he chose to take it:

No, [laughter], I didn’t. But it was related to gambling. The cruise is something they do then, so gambling companies rent a boat and then they have a cruise and you gamble on the boat. […] And I didn’t want that when I had quit gambling. (42-year-old man, lifetime GD).

There were different coping experiences among participants with current GD. Again, this seemed to be related to how psychologically distanced they were to gambling overall. One participant was still actively gambling and did not speak of any active coping efforts, although he conveyed negative attitudes toward direct marketing. Another gambler had recently started to deal with his problems and stated that the marketing influence was dependent on whether or not he was in “resistance mode” or “play mode.” The third had been abstinent for a few weeks and stated that direct marketing could still trigger urges to play, although he resisted by focusing on the negative consequences of gambling. The fourth had stopped gambling for a few months. Despite easily resisting the more common promotions, he anticipated that information about potential large-scale gambling events with attractive prizes would pose a challenge. The last participant had been abstinent for several months and did not speak of much current influence.

When considering concrete strategies, the most frequent was to either attempt to exclude oneself from the gambling company/site or to dispose of the emails, letters, and texts as soon as they came. This was done by those with lifetime GD, because they would continue to receive direct marketing, as well as those with current GD. Those who spoke of actively excluding themselves described this as a cumbersome process and one that was ineffective in handling direct marketing from unknown gambling sites/companies and, as noted above, even from some of those sites that were frequently visited. A few noted that they ended up opening a new email account in order to avoid direct marketing.

## Discussion

This exploratory study was conducted with broad research questions in order to capture as many experiences related to gambling-related direct marketing as possible. The results showed that the participants had varied experiences related to all aspects of the research questions, including types of direct marketing and corresponding attitudes, influences, and interactions. Two key findings in the present study were that the marketing experiences were intimately connected to the participants’ overall gambling behavior as well as to their relationships to their own gambling problems and that direct marketing was an interactive form of marketing, both in itself and through the promotions it contained.

### Types of Direct Marketing and Attitudes

The participants’ experiences with different types of direct marketing map onto several trends in the use of marketing techniques and the gambling market overall. The participants experienced direct marketing that was primarily related to online gambling providers outside Norway (except for two participants who had consented to and received lottery reminders from Norsk Tipping) because state-regulated providers in Norway face strong restrictions on direct marketing (Ministry of Agriculture, 2014). The close connection between direct marketing and online gambling manifested itself in two ways. Direct marketing was conducted through online channels and was used to direct the potential gambler to online gambling opportunities. Direct marketing by email was perceived as especially well-known and generic, which has its own set of implications. While mass communication and personal communication (i.e., direct marketing) have been conceived of as separate (Van Waterschoot and Van den Bulte, 1992), some of the experiences found in the present study suggest some overlap. Even if emails and texts can be sent directly to one recipient, they can also be routinely and automatically sent out to several costumers at once, and the participants in the current study often experienced them as being automatically generated and impersonal.

All participants had experienced promotional marketing such as free spins and bonuses, and the participants’ attitudes toward these promotions were somewhat similar to the findings reported by Thomas et al. (2012) who investigated attitudes toward gambling marketing among problem and non-problem gamblers. Like those with current problem gambling in that study, participants in our study stated that they used to appreciate promotions during active play periods. The present attitudes toward direct marketing among participants with lifetime GD in our study were in line with the general findings by Thomas et al. (2012) there was a perceived oversaturation in marketing and promotional marketing as well as a perception that the marketing was deceitful to varying degrees. Furthermore, in the present study the attitudes of those with current GD were more varied, with some statements suggesting that the direct marketing offers was seen as immoral, whereas others were more concerned with the attractiveness of offers. A preoccupation with the personal gains related to the offers suggests less psychological distance from gambling, i.e., being at an early stage of changing gambling behavior (Prochaska and DiClemente, 1986; Petry, 2005).

Access and availability can be understood as prerequisites for the development of GD (Blaszczynski and Nower, 2002). The participants’ experiences with direct marketing types appear relevant to both. Availability for both known and unknown gambling companies was conveyed through direct marketing in a way that was experienced as overwhelming and aggressive by the participants. In terms of access, the combination of direct and promotional marketing seems important. Similarly, to the study by Hing et al. (2014) it was found that promotions were tailored to participants’ gambling patterns and were based on the individual’s overall gambling intensity with a company and their current level of activity/inactivity. While gambling intensively, typical marketing was characterized by bonuses and cash back agreements, whereas marketing targeted at currently inactive gamblers was characterized by free credits. Better and personalized bonuses and cash back agreements as well as substantial amounts of free credit suggest that direct marketing is important for motivating increased expenditure at the site and to encourage gamblers who have reduced or stopped their gambling to resume.

The finding that some participants received free credits when they were inactive could be regarded as intrusive. Such intrusions may interfere with what would otherwise be a natural pause in the gambling involvement, including both momentary distancing and possible reflection over one’s own behavior, which is seen as an important stage in changing gambling behaviors (Petry, 2005; Derevensky et al., 2010). The experiences captured in the subtheme “Direct marketing as predatory” showed that some participants were induced to return to gambling even when they had attempted to exclude themselves. Hing et al. (2014) also report some cases of promotions through emails and texts being used to attract former gamblers. In the present study, these experiences involved email promotions but also phone calls that involved money/free credit offers or appeals for dialog and negotiation.

### Influences and Interactions With Direct Marketing

Participants reported that direct marketing had influenced them by increasing their gambling. The effect on their gambling was generally described as greater than for indirect marketing, with some explaining that the tailoring and personalization of the direct marketing made them feel special. One could argue that this feeling might increase a sense of commitment to gambling and perhaps to the specific gambling company as well. Direct marketing triggered gambling urges and induced conflicts between gambling and control behavior, as described in the subtheme “Becoming part of the problem.” The subtheme “Disengaging with direct marketing” highlighted coping strategies but also concerned how direct marketing continued to exert an influence during more concentrated efforts on behalf of the participant to stop gambling. Participants with current GD who tried to stop gambling reported direct marketing to interfere with this, while participants with lifetime GD or longer periods of abstinence mostly denied any present influence from marketing overall. In terms of the stages of change model, this suggests that direct marketing may exert an influence during the action stage but not during the maintenance stage (Petry, 2005).

The current findings are comparable to those of previous studies but are also novel in certain respects. Similar to Binde (2008) it was found that participants reported an influence from marketing because it triggered gambling urges and increased involvement. However, the influence was described as more extensive in the present study compared to what was reported in Binde (2008) study, which might be due to a focus on basic marketing in Binde (2008) study. This explanation is supported by studies suggesting stronger influences from promotional marketing compared to basic marketing (Hing et al., 2014; Deans et al., 2017).

Interaction and influence from direct marketing were closely connected. Experiences captured in the subthemes “Using and assisted by direct marketing” and “Becoming part of the problem” indicated that direct marketing became part of the actual gambling behavior, in addition to being something that influenced it. Participants would make different use of offers, and either manipulated how they occurred or made use of them as they were offered. Regardless, they took an active role when interacting with them. The interaction was 2-fold, involving both direct communication with gambling companies and the use and manipulation of the promotions being offered. Direct marketing enabled continual exchanges between the companies and the gambler. Promotional marketing afforded more opportunities for interaction compared to basic marketing, for example, bonus offers might be differently acted upon. It was found that participants used promotions to enable and extend gambling sessions, similar to what was reported in Hing et al. (2014). Some participants stated that they would make use of free spins more often when they were out of gambling funds and would make use of bonus offers when they wanted to extend their gambling funds. These accounts of how gamblers interacted with promotions provide further support for the role of promotions in increasing access to gambling. In addition, and as suggested by Hing et al. (2014) they might also be understood as reinforcement of gambling behavior and thus as facilitating addictive development (Blaszczynski and Nower, 2002).

The current findings can be interpreted within a cue-reactivity framework. Tricker et al. (2016) found that increased cue-reactivity is mediated by an altered state of consciousness, which they suggest not only elicits urges, but also elicits cognitive biases that are normally active under gambling (Toneatto, 2002). Such an interpretation might explain the findings from previous studies suggesting that GD are more influenced by promotional marketing compared to non-problem gamblers, which is explained by those with GD viewing the offers as more attractive due to cue-activated cognitive biases. In the present study promotions were seen as attractive during active play and as trickery when the participant had been abstinent for a long period. Direct marketed promotions might be special in the sense that personalized offers and an emphasis on customer relations can potentially strengthen the illusion of control/skill (Toneatto, 2002). Getting the “special treatment” might be seen as evidence for one’s own success or as a signal that continued gambling involvement might eventually pay off.

A cue-reactivity framework is also applicable in explaining the experiences captured in the subtheme “Disengaging with direct marketing.” Participants who had been abstinent for long periods of time noted how marketing lost its significance, and they had more negative attitudes toward the offers, thus implying that the role of marketing as a discriminative stimulus had changed. Understood in this way, longer periods of abstinence might weaken cue-reactive responses, which will not only reduce urges but also avoid the activation of cognitive biases. The participants with current GD also reported different levels of influence, which appeared to be dependent on the amount of time the participant had been abstinent.

### Implications for Treatment and Regulation

The participants reported exposure to vast amounts of marketing, and the marketing was perceived as having a negative influence on their coping. This suggests that treatments for GD should address marketing. Cognitive behavioral therapy is currently the most well-established treatment model for GD (Cowlishaw et al., 2012) and employs both exposure techniques and cognitive restructuring, sometimes combining the two (Tolchard, 2017). While simple exposure might be effective in reducing the influence of basic marketing, it may be that a combination of cue-exposure and cognitive restructuring is necessary for changing beliefs, urges, and behaviors related to promotional marketing. Such interventions could then be conducted during treatment sessions by accessing and challenging related beliefs when viewing the promotions. However, homework in terms of planned exposure to direct marketing stimuli coupled with response prevention could also be included (Blaszczynski et al., 2005). Direct marketing should also be a topic for relapse prevention sessions in therapy as gamblers may continue to receive direct marketing after they have quit gambling.-The findings also have implications for policy makers. It has been found that users of online gambling sites report promotional marketing such as bonuses and free credit to be an important reason for gambling on the sites (Gainsbury, 2012). In the present study, promotions such as these were heavily tailored and directly marketed to the individual. On account of being unregulated in Norway, these online sites do not face the same restrictions on marketing as licensed gambling companies. The apparent influence of marketing on GD makes the lack of regulation of some gambling companies’ marketing problematic, and this is especially so when considering that regulation of online marketing has been found to be significantly associated with reduced rates of GD (Planzer et al., 2014). The influence of direct marketing reported here needs to be confirmed in future studies, but the existing research quite consistently suggests that direct marketing should be of interest to policy makers because it potentially has a stronger impact on gamblers than indirect marketing.

## Limitations, Further Research, and Conclusion

Sample characteristics could be regarded as a limitation of the study because they precluded the investigation of some experiences that could be an interesting inquiry for future research. Among these are experiences related to other stages of change (Hanss et al., 2015). It is possible that different experiences are present among people with GD who do not yet see themselves as having a problem – those in the pre-contemplation stage. The perceived effects of accepting direct marketing involving events such as sponsored trips, dinners, and parties could not be investigated in the present study because none of the participants had accepted such offers. It is therefore still unknown how attendance at such events is experienced by people with GD. The sample was also limited by containing just two women.

The experiences in the current study are likely to resonate with gamblers beyond the study sample, termed transferability in qualitative research (Tracy, 2010). The participants in the current study experienced direct marketing as attractive, intruding, and/or triggering. Similar types of experiences have been reported in relation to other types of gambling marketing (Binde, 2008; Thomas et al., 2012; Hing et al., 2014). The common nature of these types of experiences suggest that gamblers receiving direct marketing in other countries may also resonate with the experiences reported in the current study. However, questions regarding the general prevalence and frequency of exposure, the strength of the influence, and the levels of interaction are better answered through quantitative study designs. As argued by Binde (2014) the influence from gambling marketing is best understood relative to other factors, and thus investigating the influence of direct marketing while controlling for different group memberships and risk factors is warranted. Finally, a more in-depth content analysis of direct marketing could give insights into its unique aspects in terms of wording and implicated game types. Getting access to direct marketing content might be challenging because it is sent to the individual person and thus is not publicly available like television commercials, for example. One solution could be to allow participants with varying levels of gambling involvement to submit copies of emails and texts that they have received.

The present study has some notable strengths. A specific and unexplored form of marketing was examined with broad research questions, which led to the identification of varied experiences. The study provides insight into particularly intense marketing experiences among a group that has previously been found to be more influenced by gambling marketing than other gamblers. Furthermore, this study provides insights into how marketing experiences might vary depending on stages of change.

## Data Availability Statement

The datasets generated for this study will not be made publicly available. The dataset analyzed for this study is not publicly available because the participants and the ethical committee that approved the study have been guaranteed that only named researchers will have access to the dataset in order to ensure participant privacy and confidentiality.

## Ethics Statement

The studies involving human participants were reviewed and approved by Regional Committee for Medical and Health Related Research Ethics in Western Norway (No. 2017/1172). The patients/participants provided their written informed consent to participate in this study. Written, informed consent was obtained from the participants for the publication of any potentially identifiable data included in this article. Specifically, information on age, gender and gambling history and use of verbatim quotes.

## Author Contributions

AS, RM, and SP conceived and designed the study. AS conducted the data collection. AS and RM conducted the main analysis. All authors made substantial contributions to the interpretation of the data, contributed to manuscript revision, read and approved the submitted version.

## Conflict of Interest

The authors declare that the research was conducted in the absence of any commercial or financial relationships that could be construed as a potential conflict of interest.
